# Enhancement of Gas Barrier Properties and Durability of Poly(butylene succinate-co-butylene adipate)-Based Nanocomposites for Food Packaging Applications

**DOI:** 10.3390/nano12060978

**Published:** 2022-03-16

**Authors:** Astrid E. Delorme, Tanja Radusin, Petri Myllytie, Vincent Verney, Haroutioun Askanian

**Affiliations:** 1 CNRS, Clermont Auvergne INP, ICCF, Université Clermont Auvergne, 63000 Clermont-Ferrand, France; vincent.verney@uca.fr; 2 Norner Research, Dokkvegen 20 NO-3920, 3920 Porsgrunn, Norway; tanja.radusin@norner.no (T.R.); petri.myllytie@norner.no (P.M.)

**Keywords:** biodegradable polymers, food packaging, layered double hydroxides, nanocomposites, fillers, poly(butylene succinate-co-butylene adipate)

## Abstract

Poly(butylene succinate-co-butylene adipate) (PBSA)-based materials are receiving growing attention in the packaging industry for their promising biodegradability. However, poor gas barrier properties and low durability of biodegradable polymers, such as PBSA, have limited their wide-spread use in food packaging applications. Here we report a scalable solution to improve gas barrier properties and stabilize PBSA against photo-aging, with minimal modifications to the biodegradable polymer backbone by using a commercially available and biocompatible layered double hydroxide (LDH) filler. We investigate and compare the mechanical, gas barrier, and photoaging properties of PBSA and PBSA-LDH nanocomposite films produced on a pilot scale. An increase in rigidity in the nanocomposite was observed upon addition of LDH fillers to neat PBSA, which direct the application of neat PBSA and PBSA-LDH nanocomposite to different food packaging applications. The addition of LDH fillers into neat PBSA improves the oxygen and water vapour barriers for the PBSA based nanocomposites, which increases the attractiveness of PBSA material in food packaging applications. Through changes in the viscoelastic behaviour, we observe an improved photo-durability of photoaged PBSA-LDH nanocomposites compared to neat PBSA. It is clear from our studies that the presence of LDH enhances the lifetime durability and modulates the photodegradation rate of the elaborated biocomposites.

## 1. Introduction

The UN Environment Programme estimates that plastic pollution costs 13 billion USD in economic damage to marine ecosystems per year globally [1]. A significant proportion of this pollution originates from the packaging sector, which has in general a short ‘in-use’ lifetime of less than 6 months [2]. In parallel, food packaging is a growing global economic actor with a revenue expected to reach 411.3 billion USD by 2025 [2]. Replacing non-biodegradable polymers that are typically used in food packaging and single-use goods, such as polyethylene terephthalate (PET), polypropylene (PP) and polystyrene (PS), with biodegradable polymers presents one of the most promising routes to meet market needs and ensuring that the amount of persistent plastic debris in the environment is minimised through appropriate composting of biodegradable packaging into CO_2_, H_2_O and biomass [3,4,5,6].

Poly(butylene succinate-co-butylene adipate) (PBSA)-based materials are currently receiving attention in the packaging industry for their promising biodegradability. PBSA is synthesized from 1,4-butanediol, succinic acid and adipic acid monomers by copolymerization, which are now also popularly biosourced [2,7]. However, like many biodegradable polymers, PBSA needs appropriate fillers to meet specific processing and application requirements. PBSA is exposed to thermomechanical degradation and photodegradation during both its processing and service life which initiate and propagate changes in the chemical structure of the polymer, mainly through an oxidative route, causing deterioration of physical properties (e.g., cracking, embrittlement, and discolouration) [8,9,10,11]. For this reason, it is integral to choose a stabilization strategy which can inhibit or retard the degradation in order to avoid structural changes and ensure high durability of the polymer throughout its production and use [10].

Polymer matrices are normally reinforced with fillers in order to tailor polymer properties for specific applications. Within the field of polymer fillers, nanocomposite technology has become a very active area of research, since very small amounts of filler can lead to a significant enhancement in polymer durability and gas permeability properties as compared to those of pure polymer or the conventional composites [12,13]. Additionally, the small size of the nanofillers often enables good dispersion and high synergism between the polymer matrix and the additives. Layered double hydroxides (LDHs), or anionic clays, are now gaining attention for their use as fillers, as they display facile particle size control, tunability, and functionalization as well as a notable biocompatibility which increases their attractiveness for food packaging applications [14,15]. LDHs are a type of lamellar solids and can be represented by the general chemical formula [M^II^_1−x_M^III^_x_(OH)_2_]^x+^ A^n−^_x/n_·mH_2_O, where M^II^ and M^III^ are the divalent and the trivalent cations, respectively, A^n−^ is the exchangeable interlayer anion(s) whose function is to balance an excess positive charge induced by the metal cations [16]. Although the use of LDHs as heat stabilizers and acid scavengers in halogenated polymers (such as PVC) is well known and is used on industrial scale [17], their use in biodegradable polymers to tune the durability and gas permeability properties has only just recently been reported. For example, recent studies have shown that addition of LDH to neat PBSA can enhance mechanical and gas barrier properties as well as prolong thermal and photo-durability [18,19,20,21]. Furthermore, previous studies have reported that LDH fillers have in general a catalytic effect on the biodegradation or hydrolytic degradation of different biodegradable polyesters such as poly(lactide acid) and polycaprolactone [22,23,24]. Despite the promising prospects of developing high performing PBSA-LDH nanocomposites for food packaging applications, achieving high TRL (Technology Readiness Level) of PBSA-LDH nanocomposite production has not yet been extensively explored.

In this study, we investigate the TRL of PBSA-LDH nanocomposite production for food packaging applications by producing nanocomposite films on pilot scale (producing materials with a total mass of 12 kg at Norner Research) based on PBSA which is produced from bio-based succinic acid and 1,4-butanediol, compostable and is of food contact grade (complying to EU10/2011). We adopt the approach of mixing LDH fillers into PBSA to improve the overall durability and gas barrier properties of the nanocomposites. The commercially available hydrotalcite based SORBACID^®^ 911 is our choice of LDH- filler due to its low-cost and also because it is listed as an acceptable material in the “Plastic Food Contact Materials” report (Regulation (EU) 10/2011). Moreover, considering that SORBACID^®^ 911 is used on industrial scale as an acid scavenger for PVC materials, it already presents an appropriate scalability for food packaging applications [17]. Hydrotalcite LDH materials can be found in nature (e.g., brucite) and are also increasingly used as environmentally friendly alternatives in a range of environmental applications including the adsorption of aqueous and atmospheric pollutants [25]. The properties of PBSA-LDH based nanocomposites were investigated by rheological, tensile testing, permeability testing, and thermal and optical methods, which demonstrate that the PBSA-LDH combination forms compatible nanocomposites with good dispersion. The addition of SORBACID^®^ 911 fillers enhanced the gas barrier properties, in particular, the water vapour barrier, rendering the nanocomposites competitors to conventional plastic films used for food packaging [12]. Furthermore, the photo-durability of the PBSA-LDH nanocomposites is examined through accelerated photoaging and compared to natural weathering. The results demonstrate improved resistance to photodegradation and weathering compared to pristine PBSA, and the resistance towards both photoaging and weathering increases with the LDH concentration in the PBSA matrix. With the improved gas barrier properties together with increased durability of PBSA with added SORBACID^®^ 911, we demonstrate a realistic and scalable opportunity for transitioning from non-biodegradable to biodegradable food-packaging.

## 2. Materials and Methods

### 2.1. Materials and Composites Preparation

Poly(butylene succinate-co-adipate) (PBSA) polymer grade FD92PM was obtained from MCPP Germany GmbH (Düsseldorf, Germany), molecular weight of 516.54 g mol^−1^, melt-mass flow rate (190 °C, 2.16 kg) of 4 g (10 min)^−1^ and density of 1.24 g cm^−3^. Commercial LDH mineral SORBACID^®^ 911 was obtained from Clariant (Muttenz, Switzerland), with a density of approximately 350 g L^−1^, an average particle size (d50) of ≤1 µm and 90% of particles having a size less than 10 µm. The chemical formula of SORBACID^®^ 911 is [Mg_x_Al_2_(OH)_2(2+x)_]CO_3_ × nH_2_O, where x = 4−6 and n = 0−10 [17].

PBSA was extruded with the LDH mineral at LDH addition levels of 2 wt%, 5 wt%, and 8 wt%. The polymers were pre-blended in powder form before extrusion. The extrusion was performed using PRISM24 twin screw extruder (Thermo Fisher Scientific, Waltham, MA, USA). The process temperature was set at 160 °C, and the screw speed at 300 rpm. PBSA composites were extruded into monolayer films using laboratory-scale blown film extrusion line E 25 P from Collin (Collin Lab & Pilot Solutions GmbH, Maitenbeth, Germany). All three PBSA-LDH nanocomposite films (2, 5, and 8 wt% LDH) were produced with total mass of 12 kg (corresponding to 11.4 kg of PBSA and 0.6 kg of SORBACID^®^ 911) to yield 4 kg each of the three sets of PBSA-LDH nanocomposite films. Neat PBSA was also processed for a final product of approximately 4 kg.

### 2.2. Scanning Electron Microscopy (SEM)

SEM was used to provide information of sample surface topography/composition. SEM images were collected on a Hitachi SU 5000 with an acceleration potential of 10 kV that was equipped with a scattering electron detector/UVD. The LDH size distribution in the nanocomposite films was analyzed using open-source ImageJ software.

### 2.3. Thermogravimetric Analysis (TGA)

TGA was used to evaluate the thermostability of PBSA and PBSA-LDH films. The TGA analyses were carried out on the films with a mass of approximately 10–15 mg in a nitrogen atmosphere on a Mettler Toledo STARe TGA/DSC 1 instrument. The samples were heated from 30 °C to 700 °C, with a heating rate of 10 °C min^−1^.

### 2.4. Differential Scanning Calorimetry (DSC)

DSC was used to determine temperature as well as the enthalpy of melting and crystallization of PBSA and PBSA-LDH films. A Mettler Toledo STARe DSC 3+ instrument (Columbus, OH, USA) was used for the DSC analyses under an air flow, the samples with a mass of roughly 4–5 mg were subjected to a heating step from ambient temperature to 180 °C, then a cooling step from 180 °C to ambient temperature and finally a second heating step from ambient temperature to 180 °C. All steps were conducted with a rate of 10 °C min^−1^. The degree of crystallinity (*X_c_*) is defined by the ratio between the experimentally measured melting enthalpy of PBSA (Δ*H_m_*) and the theoretical melting enthalpy of 100% crystalline PBSA ($\Delta H_{m}^{0}$). In the case of PBSA based nanocomposites, only the fraction of PBSA (*w*) should be taken into consideration, and then, the Equation (1) is modified as follows:

(1) $$X_{c}=\frac{\Delta H_{m}}{w\cdot \Delta H_{m}^{0}}\cdot 100\left[\%\right]$$

Here, the theoretical melting enthalpy of 100% crystalline PBSA ($\Delta H_{m}^{0}$) was considered to be 116.9 J g^−1^ [26].

### 2.5. Tensile and Tear Tests

Tensile tests of PBSA and PBSA containing LDH films were conducted according to ASTM D638 and ISO 527 using an Instron mechanical machine MTS 200. The sample films were cut into the dumbbell shape according to Type 2 with a 25 mm length, 4 mm width and 64 ± 7 µm thickness. The samples were tested in transverse direction (TD) and machine direction (MD) at room temperature, with a testing speed of 30 mm min^−1^. The tests for each film were repeated at least five times to determine the standard deviations.

### 2.6. Rheology

The viscoelastic properties of PBSA and PBSA based nanocomposites and changes in the molecular structure of PBSA and PBSA-LDH polymer films as they were aged in various conditions were monitored by melt viscoelastic experiments in oscillatory shear mode using a rotational controlled strain rheometer (ARES/Rheometric Scientific, manufactured by TA Instruments, New Castle, DE, USA) equipped with parallel plates geometry. The diameter of the plates was 8 mm, and the gap between the plates was approximately 1 mm. A strain amplitude of 5% was applied to ensure that all measurements were conducted within the linear viscoelastic region. A frequency sweep extending from 0.1 to 100 rad s^−1^ was performed at 110 °C.

### 2.7. Infrared Spectroscopy

Fourier-transform infrared spectroscopy (FT-IR) was used to investigate the possible changes of functional groups within PBSA and the PBSA nanopcomposites with LDH loadings. The spectra of the films were recorded between 4000 cm^−1^ and 400 cm^−1^ with a resolution of 4 cm^−1^ and 32 scans, on a Nicolet 6700 FT-IR Themo Scientific (Madison, WI, USA).

Attenuated total reflectance infrared spectroscopy (ATR-IR) was used to record the infrared spectrum of the SORBACID^®^ 911 powder. The spectrum was recorded between 4000 cm^−1^ and 400 cm^−1^ with a resolution of 4 cm^−1^ and 64 scans, on a Nicolet 380 IR Thermo Electron Corporation (Madison, WI, USA) with a Golden GateTM Single Reflection Diamond ATR SPECAC.

### 2.8. Gas Barrier Properties

#### 2.8.1. Water Vapor Transmission Rate

The water vapor transmission rates (WVTR) of the films were determined using a water vapor permeation analyzer Permatran-W 3/34 (Mocon, Minneapolis, MN, USA) that complies with ISO 15106-2 standard. The WVTR test was performed at 37.8 °C and 100% relative humidity for 24 h. Two circular specimens were cut from the films and mounted in the two parallel chambers of the apparatus. Humid test gas is flushed on one side of the barrier material, permeates through the material to dry carrier gas on the other side of the material, and is flushed to an infrared water vapor sensor. The steady-state WVTR data were collected for both chambers, and the mean values were calculated. The test was repeated twice for PBSA and each PBSA-LDH nanocomposites.

#### 2.8.2. Oxygen Transmission Rate

The oxygen transmission rates (OTR) of the films were measured with an OTR Permeability tester Mocon OX-TRAN 2/22 H (Mocon, Minneapolis, MN, USA) that complies with ISO 15105-2. The OTR test was performed at 23 °C and 0% relative humidity. Two circular specimens were cut from the films and clamped into the two parallel chambers of the apparatus. Test gas with known oxygen content is flushed on one side of the barrier material, permeates through the material to oxygen free carrier gas on the other side of the material, and is flushed to a coulometric oxygen sensor. The test was repeated twice for PBSA and each PBSA-LDH nanocomposites.

### 2.9. UV–VIS Spectroscopy

UV–VIS spectroscopy was used to monitor yellowing of the films as they were aged. The spectra of the aged and non-aged films were recorded between 800–200 nm with a resolution of 0.5 nm on a UV-2600 Shimadzu (Kyoto, Tokyo). Solid state UV–VIS of SORBACID^®^ 911 powder was obtained on a Shimadzu UV-2600 in a range of 200–500 nm with BaSO_4_ background.

### 2.10. Accelerated Photoaging

PBSA films with and without LDH were exposed to UV irradiation at 60 °C in dry air in an accelerated photoaging chamber (based on SEPAP 12–24 device which is described elsewhere [27]). The chamber emits polychromatic radiation and is equipped with a ‘medium pressure’ mercury source filtered by borosilicate envelope (Mazda type MA 400) supplying radiation of wavelengths longer than 300 nm. The mercury source is located along the focal axis of a cylinder with elliptical base. Sample films, fixed on aluminum holders, turned around the other focal axis. The inside of the chamber is made of highly reflecting aluminum. The temperature of the films was controlled by a thermocouple connected with a temperature regulator device which controls a ventilator. Films were taken for analysis after various exposure times in the SEPAP.

### 2.11. Natural Weathering

Natural weathering of PBSA and their nanocomposites films was conducted at Clermont-Ferrand (France, latitude 45_450 N and longitude 3_100 E, altitude 394 m) for four months, from March to July 2021. The films were fixed on aluminum holders mounted on 45° south facing. The films were subjected to daily sunlight, and samples were taken periodically for analysis.

## 3. Results and Discussion

### 3.1. Initial Characterization of PBSA and PBSA-LDH Nanocomposite Films

The polymer backbone and the possible interactions between the incorporated LDH fillers within the polymer can influence the overall properties of the polymer material (viscoelastic, thermal, optical, mechanical, etc.) and consequently can lead to modification of the process (film extrusion, blow or injection molding) used to prepare the final product. In the following section, the thermal and mechanical properties of PBSA-LDH based nanocomposites are investigated and compared to the properties of neat PBSA.

#### 3.1.1. Microscopy of Films

**Figure 1:**
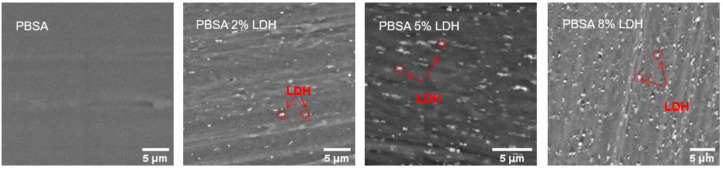
SEM images collected of PBSA and PBSA nanocomposite films with 2 wt%, 5 wt%, 8 wt% LDH demonstrating the dispersion of the LDH particles in the films.

The overall bulk gas barrier and mechanical properties of nanocomposite materials polymer film are, typically, strongly influenced by the morphology and dispersion of the nanoparticles in the final product after processing [3,28]. The LDH dispersion in PBSA based films was evaluated by SEM and Figure 1 shows SEM images of PBSA and PBSA nanocomposite films containing 2 wt%, 5 wt%, and 8 wt% LDH.

As observed in the SEM images in Figure 1, the LDH particles are well dispersed in all samples. Furthermore, the size distribution of dispersed LDH particulates in the PBSA matrix was evaluated by ImageJ software (size distribution graphs are shown in Figure S4 in the Supplementary Materials), which showed that LDH size distribution was equal to the average SORBACID^®^ 911 particle size (d50) of ≤1 µm, confirming that no LDH agglomerates are present in the PBSA nanocomposite films. It can therefore be concluded that the compounding extrusion was successful in dispersing LDH fillers into PBSA polymers with up to 8 wt% LDH loading on pilot scale production.

#### 3.1.2. Thermal Properties

**Figure 2:**
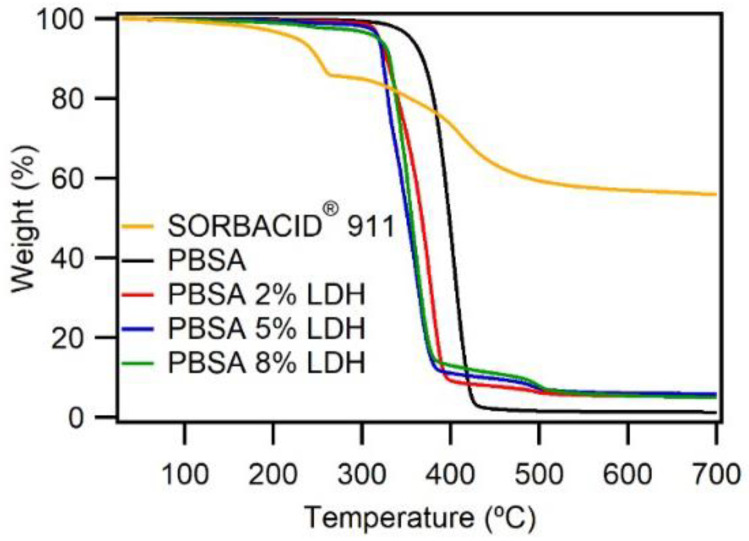
TG curves of SORBACID^®^ 911 (yellow line), PBSA (black line), and PBSA-LDH nanocomposites (red line 2%wt LDH; blue line 5%wt LDH; green line 8%wt LDH). The samples were heated from 30 °C to 700 °C, with a heating rate of 10 °C min^−1^.

Thermal properties of polymers and nanocomposites are important to investigate in order to evaluate their appropriateness for the large-scale processing at an elevated temperature [24]. The thermal properties of PBSA films were characterized by means of TGA and DSC. Figure 2 shows the TGA curves of SORBACID^®^ 911, PBSA, and PBSA-LDH nanocomposites, and Table S1 in the Supplementary Materials summarizes the thermal decomposition data of the curves.

In general, the thermal decomposition of LDHs occurs in three different steps; there is the first step of evaporation of interlayer and absorbed water molecules (below 250 °C), this is then followed by the second step which is the loss of the metal hydroxide layer (250–360 °C), and finally, the third step is the decomposition of the interlayer organic anion (330–500 °C) [29,30]. An overlap between the second and the third decomposition mass loss is commonly observed, which is also reflected in Figure 2 (yellow line), where the sharp first loss step (water evaporation) is followed by a broader mass loss step (loss of metal hydroxide layer and decomposition of the interlayer organic anion) [29,30]. In contrast, the TG curve of neat PBSA shows only one major mass loss step in the temperature range of 350–420 °C. This key mass loss event corresponds to the pyrolysis of the PBSA molecular chains [31]. As observed in Figure 2, the introduction of LDH to the PBSA matrix decreases the temperature at which the major mass loss step occurs (extrapolated from 1st derivative of the mass loss step) from 402 °C for neat PBSA to 377, 360, and 355 °C for PBSA nanocomposite films with 2, 5, and 8 wt% LDH, respectively, which suggests that the introduction of LDHs to neat PBSA lowers the thermal stability of the nanocomposites. Lower thermal stability due to addition of LDH in polymer composites has previously been reported and has been explained to be a result of the catalysed degradation of PBSA by the metal ions and the water molecules contained between the layers of the LDH which are released during the decomposition of composites [29,30,32]. Although the addition of LDH lowers the thermal stability of the nanocomposites, the PBSA-LDH nanocomposites could be processed into films on pilot scale at 160 °C for a short time period, which is significantly lower than the major degradation temperature. Furthermore, the thermal stabilities of the PBSA nanocomposites do not vary significantly between 2, 5, and 8 wt% LDH loadings. The residual mass at 700 °C for the PBSA nanocomposites observed in Figure 2 correspond to the residual LDH filler loading [29].

DSC was used to evaluate the effect of LDH loading on the melting and crystallization behaviour of PBSA. Table 1 summarizes the DSC results of PBSA and PBSA-LDH nanocomposites, where the melting temperature (*T_m_*) and the enthalpy of fusion (∆*H_m_*) were measured from the 2nd heating scan (the first heating scan served to eliminate the previous thermal history of the films). The crystallization temperature (*T_c_*), was taken from the cooling scan.

As observed from the DSC analyses in Table 1, there is almost no modification in the melting temperature of all the nanocomposites compared to the virgin matrix, while the addition of LDH into neat PBSA decreases to some degree the crystallization temperature of the overall nanocomposite. In parallel, the degree of crystallinity decreases very slightly when introducing LDH particles into neat PBSA. The lower *T_c_* and *X_c_* suggest that there is no nucleating effect of LDH in the polymer matrix, but the LDHs are rather obstacles for the polymer chains to initiate crystallization growth [20]. Changes in the crystalline properties of nanocomposites can implicate changes in the overall mechanical and gas barrier properties, which are investigated in the following sections.

#### 3.1.3. Infrared Spectroscopy

**Figure 3:**
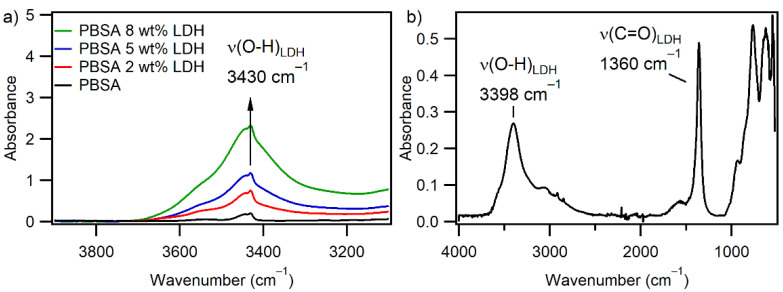
FT-IR spectra in the O-H region of (**a**) PBSA (black line) and PBSA with 2 wt% (red line), 5 wt% (blue line) and 8 wt% (green line) LDH. The peak at 3430 cm^−1^ increases with increasing loading of LDH and corresponds to the hydroxides in SORBACID^®^ 911 whose spectrum is shown in (**b**).

Figure 3a shows the FT-IR spectra of PBSA without LDH and nanocomposites with 2, 5, and 8 wt% LDH loading. PBSA has infrared absorption bands in the 2960 cm^−1^ and 2860 cm^−1^ regions, corresponding to the asymmetric and symmetrical stretching of CH_2_ groups, respectively. The most intense peak at 1740 cm^−1^ is characteristic of the carbonyl, C=O, in the ester group of PBSA [18]. Figure 3b shows the ATR spectrum of SORBACID^®^ 911, and the broad characteristic band at 3398 cm^−1^ can be ascribed to the stretching vibrations of the O−H groups on the hydroxides of the LDH; the band at 1360 cm^−1^ arises as result of the anti-symmetric stretching of the carbonates, CO_3_ [33,34,35]. The IR analysis did not demonstrate the appearance of any new bands the IR spectra of the composites are overlapping with the spectrum of the pure components (full spectra are shown in the Supplementary Materials), and it seems that no relevant reaction took place between the PBSA and LDH reactive groups. 

The broad OH band is also apparent in the PBSA-LDH films and increases with the increasing LDH loading in the film; see Figure 3a. However, the hydroxide band of the LDH is redshifted by 32 cm^−1^ when incorporated in the PBSA, which can be attributed to the formation of hydrogen bonds between the hydroxyl groups of LDH with the terminal hydroxyl groups and backbone ester groups of PBSA matrix [36].

#### 3.1.4. Mechanical Properties

The effect of introducing LDH filler into the polymer matrices on the overall mechanical properties of the PBSA films was investigated by conducting tensile tests (machine and transverse direction) on PBSA and PBSA nanocomposite films; the test results are summarized in Table 2.

Neat PBSA shows a tensile modulus of 172 MPa and strain at break of 520% (considering tests conducted in the machine direction MD) which demonstrates the ductile nature of PBSA [37]. As observed in Table 2, the addition of LDH increases the tensile modulus for all the composites because of the reinforcing effect of the rigid inorganic filler introduced to the PBSA matrix. The tensile modulus in MD direction increases by 48%, 95% and 23% for the composites with 2, 5, and 8 wt% LDH loadings, respectively. At 2 and 5 wt% LDH loadings, the mass content of LDH particles is likely to get wet by PBSA matrix leading to a significant increase in the tensile modulus, while at 8 wt% loading the tensile modulus increase only moderately compared to neat PBSA (only a 23% increase). The increase of LDH mass content to 8 wt% may disrupt the LDH wetting leading to a disruption of the interface and then to a slight decrease in the interfacial adhesion. In the same way, the stress at break increases with LDH loading up to a 5 wt% followed by a slight decrease with an 8 wt% LDH loading. It seems that 5 wt% of LDH loading is the optimal mass loading for the elaborated composites in terms of improving the mechanical properties. On the other hand, the strain at break was significantly decreased by the addition of LDH filler leading to the decrease of PBSA ductility. Generally, the fillers are unable to withstand the forces transferred by the matrix decreasing the ability of stress propagation and increasing the rigidity of the composites. The increase in rigidity with the addition of LDH fillers is an important factor to consider for choosing the appropriate application of the nanocomposites. The interaction between the LDH and the PBSA backbone, as observed in the IR-analyses, could explain considerable improvement of the elastic modulus of the nanocomposites. However, the increase of LDH content may lead to some aggregation reducing the free surfaces of LDH, and therefore, the hydrogen bonding degree could be decreased which explains the slight decrease of modulus at 8% of LDH loading. The presence of some aggregation and voids may transform the materials into brittle behavior (in addition to the fillers being unable to withstand the forces transferred by the matrix decreasing the ability of stress propagation and increasing the rigidity of the composites). Some other authors already observed similar behavior: a decrease in elongation at break by adding a small rate of inorganic filler. Han and coworkers [38] found that adding Silica above 3.5% to PBS matrix makes the elaborated nanocomposites very brittle and the tensile properties could not be measured. These mechanical properties will influence the processability of the material, which can determine the possible end-product applications. For example, higher rigidity of the end-product gives better printing properties.

Polymers that have been subjected to thermo-mechanical processing typically possess anisotropy, and this is usually revealed in differences of tensile testing data when conducting the testing in parallel or perpendicular to the machine direction of the polymer process. Such test results are important to consider when designing the final polymer product for different applications [39]. Overall, the tensile modulus and stress and strain at break in transverse direction TD have a very similar trend compared to the results of MD. The addition of LDH to PBSA did not introduce any significant anisotropic behaviour in the nanocomposite, except for the composite with 5 wt% LDH loading. At a loading of 5 wt% LDH, it seems that the tensile modulus is relatively high in MD (335 MPa) compared to the modulus measured in TD (272 MPa) suggesting that the fillers could be more oriented in MD.

#### 3.1.5. Rheological Properties

By using melt rheology, we can measure the viscoelastic properties and microstructural changes of polymers and get an insight into the modifications that are occurring on a molecular level as the LDH fillers are added to the pristine PBSA polymers. It is well-known that the zero shear viscosity, *η*_0_, depends on the molecular weight (*M_W_*) and obeys a power law as described in Equation (2) [8,9].

(2) $$\eta_{0}\propto M_{W}^{\propto }$$

**Figure 4:**
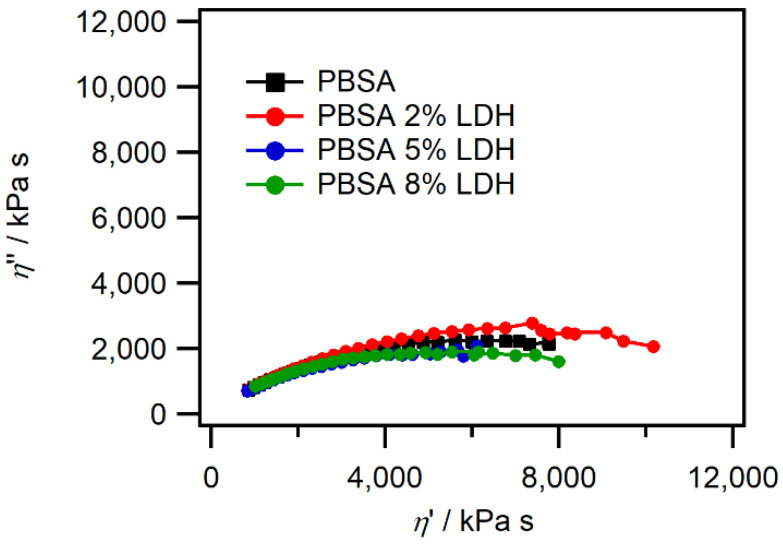
Cole–Cole plots of PBSA and PBSA-LDH nanocomposite films, obtained from melt viscoelastic rheology measurements conducted at 110 °C.

The *η*_0_ is obtained through the extrapolation of a Cole–Cole plot which predicts the dependencies of the viscosity components (*η*″ versus *η*′) to be an arc of circle in the complex plane (see Figure S10 in the Supplementary Materials). Figure 4 shows the Cole-Cole plot of PBSA and PBSA-LDH nanocomposite films with 2, 5, and 8 wt% LDH loading.

As observed in Figure 4, the Cole-Cole plots of PBSA and PBSA composites are almost overlapping, and the addition of LDH into the polymer matrix does not introduce any significant modifications in the viscoelastic properties of PBSA. This suggests that the influence of LDH fillers on PBSA plasticizing (decrease of the semicircle toward smaller values) or chain extension (increase of semicircle toward higher values) is negligible. Contrastingly to the tensile tests, the viscoelastic properties did not demonstrate any important modification in the viscoelastic properties. It should be recalled that the melt rheology analyses and tensile testing were conducted at 110 °C and ambient temperatures, respectively, and these two different temperatures could evoke different PBSA-LDH interactions. For example, it could be envisioned that the higher temperature during the melt rheology analyses decreases the hydrogen bonding interactions between LDH and PBSA, which can explain the increase in tensile strength at ambient temperature and the very limited viscoelastic variation in the melt rheology analyses conducted at elevated temperatures. Nonetheless, having similar viscoelastic properties as neat PBSA confirms that the same process parameters used for PBSA processing can be used for PBSA-LDH nanocomposites.

#### 3.1.6. Barrier Properties

**Figure 5:**
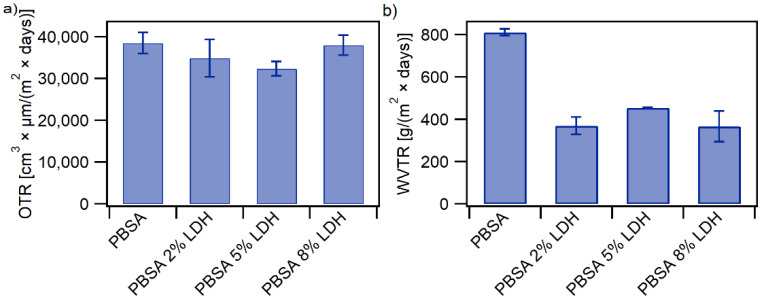
Gas barrier properties of PBSA and PBSA-LDH nanocomposite films showing (**a**) Oxygen Transmission Rates (OTR) and (**b**) Water Vapour Transmission Rates (WVTR) of PBSA films and PBSA nanocomposites with 2, 5, and 8 wt% LDH loadings. Each test was conducted in duplicates; the results show the average of the tests together with the standard deviations.

The determination of the barrier properties of a polymer is essential to predicting the shelf-life of the food-product kept within the plastic packaging. Water vapour and oxygen are two of the main permeants studied in packaging applications because they may transfer from the internal or external environments through the polymer package wall, resulting in a continuous change in product quality and shelf-life [2,40]. The OTR and WVTR for PBSA and PBSA nanocomposites with 2, 5, and 8 wt% LDH loadings were measured and are presented in Figure 5a,b, respectively.

The OTR of neat PBSA is 3.8 × 10^4^ cm^3^ µm (m^2^ day)^−1^ which is in accordance with previous published work [6,18,28,31,40]. As observed in Figure 5a, the addition of LDH into the polymer film obstructs the diffusion of the gas molecules through the film, which therefore lowers the OTR and improves the oxygen barrier property of the film. For example, the permeability is reduced up to 9% compared to neat PBSA with only 2 wt% loading of LDH. Increasing the LDH loading to 5 wt% further decreases the permeability to 16% thus improving oxygen barrier properties. Addition of inorganic particles to the polymer matrix usually leads to improvement of barrier properties due to tortuous path of molecules of oxygen through the matrix. However, an 8 wt% LDH loading in the nanocomposite showed only a very slight decrease in OTR compared to neat PBSA. A higher content of LDH increases the possibility of aggregation and void formations, leading to increased permeability of gaseous molecules through the films and therefore having a detrimental effect on the barrier properties, which has been reported by previous studies [2,6,13]. Although larger agglomerations were not detected in our SEM analyses for nanocomposite with 8 wt% LDH loading, the observed results could be a result of undetected agglomerates. These initial results, nonetheless, demonstrate good improvement of oxygen barrier properties with 2 wt% and 5 wt% LDH loading in PBSA nanocomposites. As a comparison, the OTR of mono-polymer films commonly used in food packaging is 0.1 × 10^4^ − 0.5 × 10^4^ cm^3^ µm (m^2^ day)^−1^ for PET, 5 × 10^4^ − 10 × 10^4^ cm^3^ µm (m^2^ day)^−1^ for PP and 5 × 10^4^ − 20 × 10^4^ cm^3^ µm (m^2^ day)^−1^ for PE films [12,41].

Figure 5b shows a significant decrease in WVTR upon the addition of LDH into neat PBSA and thus a noteworthy improvement in the water vapor barrier properties. Already at a loading of 2 wt% LDH, the WVTR is decreased by 55% compared to neat PBSA. Like the oxygen gas molecules, the presence of LDH particles reduces the diffusion of water molecules through the nanocomposites due to the impermeable crystalline structure of LDH resulting in a tortuous pathway for the gas molecules [15,42]. Increasing the LDH loading to 5 wt% and 8 wt% resulted in a decrease in WVTR, however to a lesser extent or almost equal WVTR to that of the nanocomposites with 2 wt% LDH. A 44% and 54% decrease in WVTR is observed for nanocomposites with 5 wt% and 8 wt% LDH, respectively which could suggest that a plateau has been reached. The improved gas barrier properties of the nanocomposites demonstrate promising prospects of using biobased and commercially available SORBACID^®^ 911 as a cost-effective, scalable, sustainable, and environmentally friendly alternative to enhance gas barrier properties of biodegradable plastic food packaging compared to the current state-of-the-art approaches of improving gas barrier properties of polymer films, including metallized films [14].

#### 3.1.7. ^®^UV–VIS Spectroscopy

**Figure 6:**
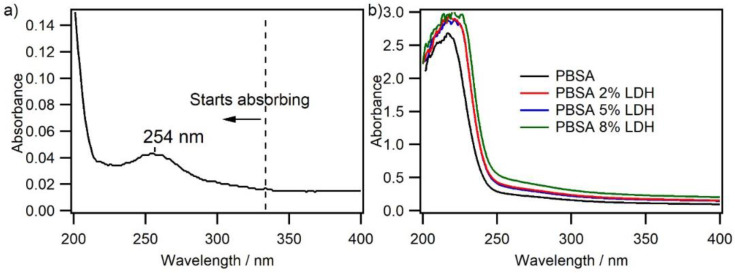
(**a**) Solid state UV absorption spectrum of SORBACID^®^ 911, and UV–VIS spectra of (**b**) PBSA (black line) and PBSA with 2 wt% (red line), 5 wt% (blue line), and 8 wt% (green line) LDH.

The UV spectrum of the LDH SORBACID^®^ 911 was recorded through solid-state UV spectroscopy and is shown in Figure 6a. Figure 6b shows the UV-absorbance of PBSA and PBSA nanocomposites with different LDH loadings.

From Figure 6a, it can be seen that, in addition to the expected light scattering, the SORBACID^®^ 911 LDH starts absorbing at a range of wavelength below 335 nm, and therefore absorbs UV-radiation emitted both from the solar radiation and the Hg-lamp used in the accelerated photoaging chamber (see Figure S3 in the Supplementary Materials). As reflected in Figure 6b, a relatively weak absorption of light in the range of 250–320 nm was observed for PBSA, which is attributed mainly to the absorption of the carbonyl groups in the PBSA polymer backbone beside the slight absorption of internal and external chromophoric impurities in PBSA [18,20]. Concerning the nanocomposites, there is an overall increase in UV-absorbance upon the addition of LDH into the neat PBSA, which increases with increasing LDH loading. Accordingly, PBSA composites based on SORBACID^®^ 911 are expected to be UV-stabilized. Recent studies have also reported the UV-stabilizing effect of LDH based fillers in nanocomposites [20].

### 3.2. Photo-Durability of PBSA and PBSA Nanocomposites

**Figure 7:**
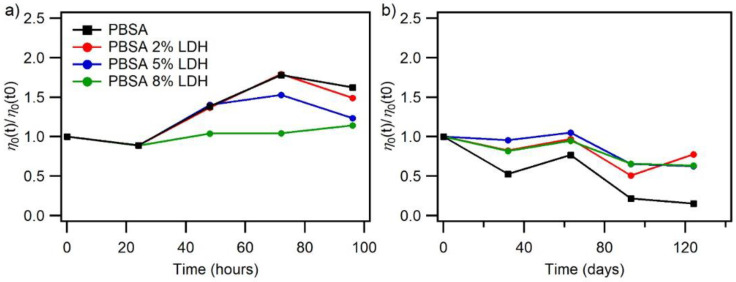
Differences in zero shear viscosity of PBSA and PBSA-LDH films as a function of exposure time in (**a**) the accelerated photoaging chamber (SEPAP 12–24) and (**b**) natural weathering.

Aging tests were conducted to test the photo-durability of PBSA and PBSA-LDH films. The photoaging of the films was realized through natural weathering and in an accelerated photo-aging chamber (SEPAP 12–24), and the samples were taken periodically for rheological and UV–VIS analyses. Photoaging is an oxidative process involving chain scission and recombination. The two mechanisms compete, and one of them is usually predominant and characterizes the overall behavior of the photoaged polymer [8,10,43]. Melt rheology is a convenient and accurate tool to study the competition of chain scissions and recombination degradation mechanisms occurring as a polymer is aged [8,9,43]. The changes in *η*_0_ can indicate whether there is a change in molecular weight of the polymer, suggesting that a transformation at the molecular level has occurred which leads to either a higher or lower molecular weight. A decrease in *η*_0_ for an aged polymer corresponds to a decrease in its molecular weight and suggests that the polymer has undergone chain scission. Vice versa, higher *η*_0_ indicates that the molecular weight of the polymer has increased and that the polymer has undergone chain recombination. The rheological analyses of the two different degradation mechanisms are explained more in detail by Commereuc and co-workers [8,9]. The measured *η*_0_ after an aging time *t* (in either the photoaging chamber or natural conditions, Figure 7a,b, respectively) normalized to the initial *η*_0_ of the unaged polymer films at time *t*0 has been plotted as a function time for each PBSA and PBSA-LDH nanocomposite films, as shown in Figure 7 (Cole-Cole plots of the aged and unaged PBSA and PBSA-LDH nanocomposites are shown in Figures S10 and S11, as well as the Dynamic viscosity relationship with frequency of unaged and aged PBSA and PBSA based nanocomposites in Figures S12 and S13).

From Figure 7a we observe an initial increase in viscosity for neat PBSA and PBSA nanocomposites with 2 wt% and 5 wt% LDH up to 72 h in the photoaging chamber which is then followed by a decrease in viscosity. The increase in viscosity suggests that there is a recombination mechanism occurring in the polymer chains, which leads to an increase in the molecular weight of the polymer. The drop in viscosity indicates that a chain scission mechanism is occurring, leading to a decrease in the molecular weight of the polymer [8,9]. Interestingly, only a slight increase in viscosity is observed for PBSA with an 8% LDH loading. The most significant variation in viscosity is observed for the neat PBSA and the degree of variation also decreases with increasing LDH loading, suggesting that the addition of LDH inhibits the degradation of PBSA as it is aged. The LDH appears to retard the degradation of the overall PBSA as it is aged in the accelerated photoaging chamber and increases the photo-durability of the overall polymer composite. As demonstrated in the UV spectrum of SORBACID^®^ 911 in Figure 6, we can see that the LDH absorbs in the UV region of the radiation emitted by a mercury lamp and part of the UV radiation emitted by solar radiation (the emission spectra of the mercury lamp and solar radiation are shown in Figure S3 in the Supplementary Materials). Thus, instead of the UV-radiation being absorbed by the polymer chains of PBSA and inducing degradative processes, the UV-radiation could mainly be absorbed by the added LDH in the composite films. The role of LDH acting as UV absorber is a likely explanation for the increasing photo-durability with increasing LDH-loading of the PBSA-LDH nanocomposites, as observed in the changes in rheological behavior over time of irradiation.

The films were also exposed to natural weathering from March to July 2021 (average daily temperature and UV readings for this period are summarized in Figures S1 and S2 in the Supplementary Materials) and Figure 7b shows the differences in viscosity of PBSA and PBSA-LDH nanocomposite films as they are aged by natural weathering. In contrast to the films aged in the accelerated photoaging chamber, an overall decrease in viscosity is observed as the films are naturally weathered. Interestingly, the different evolution of rheological properties of naturally weathered and artificially aged films suggests that the films undergo different degradative mechanisms as a result of the exposed conditions. This difference highlights the difficulty of replicating the complex nature of natural weathering in laboratory experiments. In natural weathering, there are varying conditions such as humidity, temperature fluctuations, wind forces, and varying exposure to solar radiation which can initiate and propagate various degradative reactions within the polymer chains. Notably, humidity and rain facilitate additive and degradation by-product leaching from the bulk polymer. The thermal cycling and the temperature fluctuations can either cause material contractions or expansion, support or deteriorate crystallization, which could initiate surface cracks or grazing as well as accelerate or slow down degradation mechanisms [44,45,46]. Furthermore, in the presence of moisture PBSA polymer chains are known to undergo open hydrolytic cleavage resulting in the reduction of the polymer chain length into low molecular weight oligomers, dimers and monomers [7]. The higher humidity and direct contact with rain in the natural weathering tests could be likely causes of the observed differences in natural weathering compared to the accelerated photoaging tests with controlled temperature and dry conditions. It is important to note that, again, the most significant change in the viscosity is observed for the neat PBSA matrix, the zero shear viscosity decrease by 85% after 124 days of natural weathering while the degree of variation in zero shear viscosity decreases with the LDH loading. In addition to LDH acting as a UV absorber leading to a decrease in the photoaging rate, LDH could also decrease the hydrolytic degradation rate during natural weathering. As observed in Figure 5b the addition of 2 wt%, 5 wt% and 8 wt% LDH to neat PBSA decreases the WVTR by 55%, 44% and 54%, respectively, this decrease indicates that the permeation of water molecules through the nanocomposites films containing is significantly reduced by the presence of LDH. The restriction of water molecules permeating the films induced by the LDH is likely to hamper or slow down the hydrolytic degradation of the PBSA polymer backbone chains and therefore also stabilizing the nanocomposites against hydrolytic degradation. Puchalski et al. observed a decrease in the molecular weight in accelerated conditions when they exposed PBSA matrix to different cycles of UV exposure and artificial rainfall with demineralized water demonstrating that both cycles are important to reflect the natural weathering in accelerated conditions [45]. Nevertheless, as mentioned before, the fact the LDH seems to retard the degradation of naturally weathered PBSA nanocomposites is also likely to be linked to the UV-stabilizing effect of SORBACID^®^ 911.

**Figure 8:**
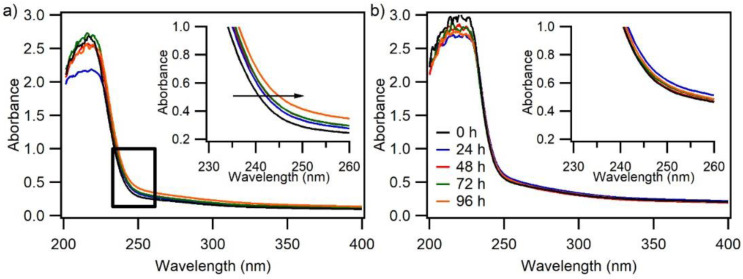
UV–VIS spectra of (**a**) PBSA and (**b**) PBSA 8 wt% nanocomposite films after being irradiated in the accelerated photo-aging chamber (SEPAP 12–24) for up to 96 h. The evolution of UV-absorbing character of the aged films over time is highlighted in the inserted graphs. All the spectra have been baseline corrected.

Figure 8a,b show the UV spectra of PBSA and PBSA nanocomposite films with 8 wt% LDH loading, respectively, after being aged in the accelerated photoaging chamber for up to 96 h. The UV spectra of PBSA nanocomposite films with 2 wt% and 5 wt% LDH loadings are shown in Figures S6 and S7 in the Supplementary Materials.

As observed in Figure 8a and highlighted in the inserted graph, which is focused on the region within the black square, there is a slight shift in the absorption of UV light of neat PBSA towards a longer wavelength. PBSA is an aliphatic polyester and lacks major chromophores in its polymer backbone, such as aromatic rings and chain conjugations. The increasing shift in UV–VIS light absorbance could be a result of carbonyl and/or conjugated degradation by-products formed as the film is aged in the photochamber [47]. Such increase in UV–VIS light absorbance upon aging is significantly hampered with the addition of LDH to PBSA, as exemplified by the UV–VIS spectra of aged PBSA nanocomposite films with 8% LDH loading in Figure 8b. A similar phenomenon is observed for aged PBSA nanocomposites with 2 wt% and 5 wt% loading and also between naturally weathered PBSA and PBSA nanocomposites, as demonstrated in their UV–VIS spectra in Figures S5–S9 in the Supplementary Materials. This observation is in accordance with the rheological results of the aging tests, in that the LDH act as a stabilizer and impedes the degradation of the polymer backbone upon aging. The stabilizing effect against chain scission and recombination reactions in polymer chains is important to consider since their polymeric structure is key for their overall properties (i.e., mechanical, optical, gas barrier properties, and biodegradability), and any alteration in its structure could be detrimental to its desired performance and consequently industrial application. The more resistant the final PBSA nanocomposite towards aging, the better durability and longer service life. This study has demonstrated the possibility of introducing SORBACID^®^ 911 fillers to both improve the gas barrier properties and photo-durability of PBSA.

## 4. Conclusions

With the growing number of global initiatives on combatting plastic pollution, such as the EU Plastic Strategy, there is a pressing urgency in investing into technologies developing biodegradable and compostable plastics for short ‘in-use’ lifetime food-packaging. To meet this market demand, a scalable solution to improve gas barrier and life-time durability of currently used biodegradable polymers is needed in food-packaging. In this study, we have successfully incorporated the biocompatible and commercially available SORBACID^®^ 911 fillers into biobased and biodegradable PBSA, with good dispersion and on pilot scale. The encouraging dual-functionality of SORBACID^®^ 911 fillers, acting both as UV-stabilizer and gas barrier moiety, offers a realistic scalable method to develop biodegradable food packaging with the necessary gas barrier properties and durability. The addition of LDH fillers increase considerably the tensile modulus of the elaborated composites and decreased the strain at break compared to neat PBSA, which should be taken into consideration when deciding on specific food packaging application, for example, applications in which rigidity is favored. Furthermore, with the facile tunability of LDH materials and inherent biocompatibility, we envision that this could unlock a range of new opportunities for the realization of tailored sustainable food packaging.

## Figures and Tables

**Table 1:** DSC results of PBSA and PBSA nanocomposites summarizing the melting temperature (*T_m_*), enthalpy of fusion (∆*H_m_*), crystallization temperature (*T_c_*), and degree of crystallization (*X_c_*_,_) analyses were conducted with a heating rate of 10 °C min^−1^.

Film	*T_m_*, °C	∆*H_m_*, J g^−1^	*T_c_*, °C	*X_c_*, %
PBSA	89.1	42.4	56.0	36.3
PBSA 2 wt% LDH	88.9	41.3	55.1	36.1
PBSA 5 wt% LDH	88.1	37.3	50.8	33.6
PBSA 8 wt% LDH	87.7	34.9	49.8	32.5

**Table 2:** Tensile properties of PBSA and PBSA-LDH films in machine and transverse directions. Testing speed used was 30 mm min^−1^.

Film	Tensile Modulus, MPa	Stress at Break, MPa	Strain at Break, %	Tensile Modulus, MPa	Stress at Break, MPa	Strain at Break, %
	Machine Direction			Transverse Direction		
PBSA	172 ± 26	20.0 ± 0.8	520 ± 16	187 ± 54	21.9 ± 3.4	561 ± 60
PBSA 2 wt% LDH	255 ± 24	24.8 ± 2.5	19.4 ± 1.2	254 ± 7	24.1 ± 0.6	17.5 ± 1.0
PBSA 5 wt% LDH	335 ± 6	29.8 ± 0.5	18.4 ± 0.6	272 ± 8	24.4 ± 0.4	16.0 ± 1.6
PBSA 8 wt% LDH	212 ± 13	19.0 ± 1.0	15.6 ± 1.5	242 ± 9	19.6 ± 1.7	13.5 ± 3.0

## Data Availability

Experimental data were deposited in the Zenodo data repository in the TERMINUS community and are publicly available at https://zenodo.org/communities/terminus-h2020/?page=1&size=20 (accessed on 11 March 2022). https://doi.org/10.5281/zenodo.6353580 (accessed on 11 March 2022).

## Supplementary Materials

The following supporting information can be downloaded at: https://www.mdpi.com/article/10.3390/nano12060978/s1, Figure S1: Average daily temperature measured at Cézeaux station during the period of sampling from March 2021 to July 2021; Figure S2: Average UV radiation measured at Cézeaux station during the period of sampling from March 2021 to July 2021; Figure S3: Emission spectrum of a Mercury lamp used in the SEPAP machine and Solar radiation arriving at earth; Figure S4: Size distribution of detected LDH particulates in SEM images; Table S1: TGA results of PBSA and PBSA composites; Figure S5 FT-IR spectra of PBSA and PBSA based nanocomposites Figure S6-S9: UV–VIS Spectra of aged and un-aged PBSA and PBSA based nanocomposites; Text 1. Melt Rheology; Figure S10: Cole–Cole plots of unaged and aged PBSA and PBSA based nanocomposites; Figure S11: Cole–Cole plots of naturally weathered PBSA and PBSA based nanocomposites; Figure S12: Dynamic viscosity relationship with frequency of unaged and aged PBSA and PBSA based nanocomposites; Figure S13: Dynamic viscosity relationship with frequency of naturally weathered PBSA and PBSA based nanocomposites.
