# Supplementary material for: Enhancement of Gas Barrier Properties and Durability of Poly(butylene succinate-co-butylene adipate)-Based Nanocomposites for Food Packaging Applications
Source: Nanomaterials (Basel). 2022 Mar 16;12(6):978. doi: 10.3390/nano12060978 (PMC8953858; doi:10.3390/nano12060978)
Supplement: Supplementary file 1 [file nanomaterials-12-00978-s001.zip › nanomaterials-1617854-supplementary.pdf]

nanomaterials

Supplementary Materials

Enhancement of Gas Barrier Properties and Durability of Poly(butylene Succinate-Co-butylene Adipate)-Based Nano-composites for Food Packaging Applications

Astrid E. Delorme [1,*], Tanja Radusin [2], Petri Myllytie [2], Vincent Verney [1] and Haroutioun Askanian [1,*]

[1] Université Clermont Auvergne, CNRS, Clermont Auvergne INP, ICCF, 63000, Clermont-Ferrand, France.CNRS , SIGMA Clermont, ICCF, Université Clermont Auvergne, 63000 Clermont-Ferrand, France; vincent.verney@uca.fr (V.V.)

[2] Norner Research, Dokkvegen 20 NO-3920 Porsgunn, Norway; ; tanja.radusin@norner.no (T.R.); petri.myllytie@norner.no (P.M.)

* Correspondence: astrid.delorme@sigma-clermont.fr (A.E.D.); haroutioun.askanian@sigma-clermont.fr (H.A.); Tel.: +33-(0)473405389 (H.A.)

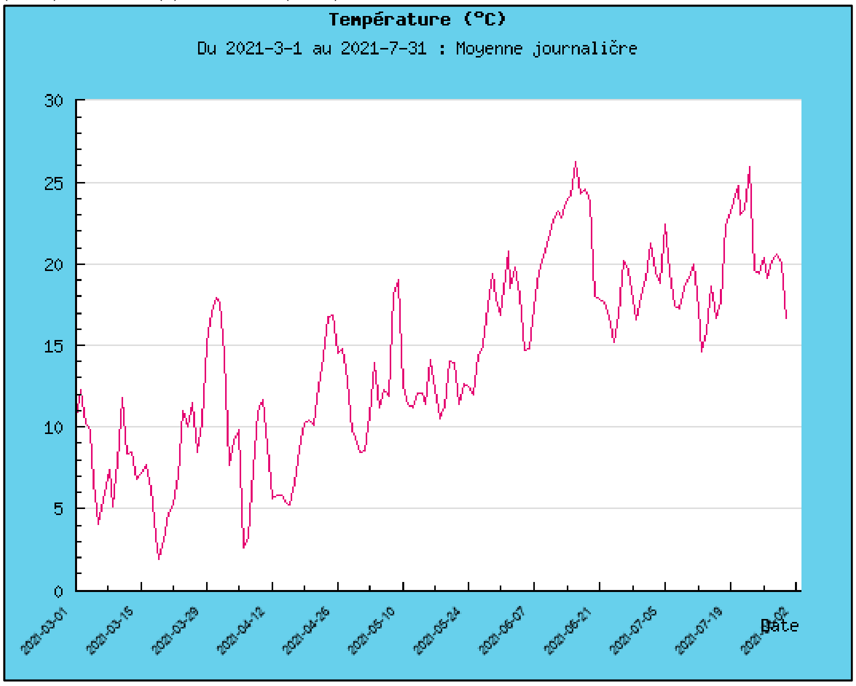

Figure S1. Average daily temperature measured at Cézeaux station (45°45'37 N – 3°06'43 E 394 m asl.) during the period of sampling from March 2021 to July 2021. The data was obtained from Observatoire de Physique du Globe de Clermont-Ferrand (http://wwwobs.univ-bpclermont.fr/SO/mesures/pdd.php) (accessed on 01/12/2021).

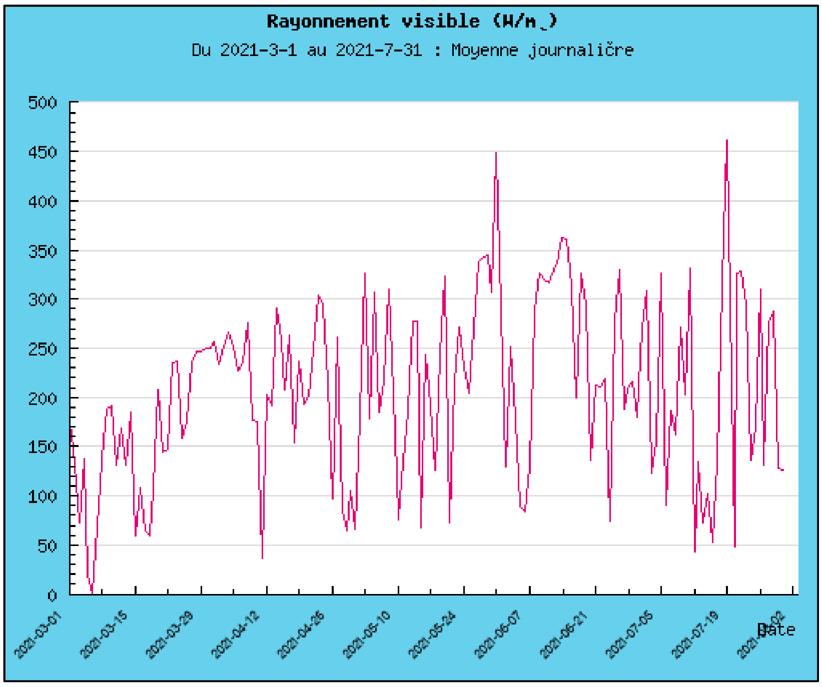

Figure S2. Average UV radiation measured at Cézeaux station (45°45'37 N − 3°06'43 E, 394 m asl.) during the period of sampling from March 2021 to July 2021. The data was obtained from Observatoire de Physique du Globe de Clermont-Ferrand (http://wwwobs.univ-bpclermont.fr/SO/mesures/pdd.php) (accessed on 01/12/2021).

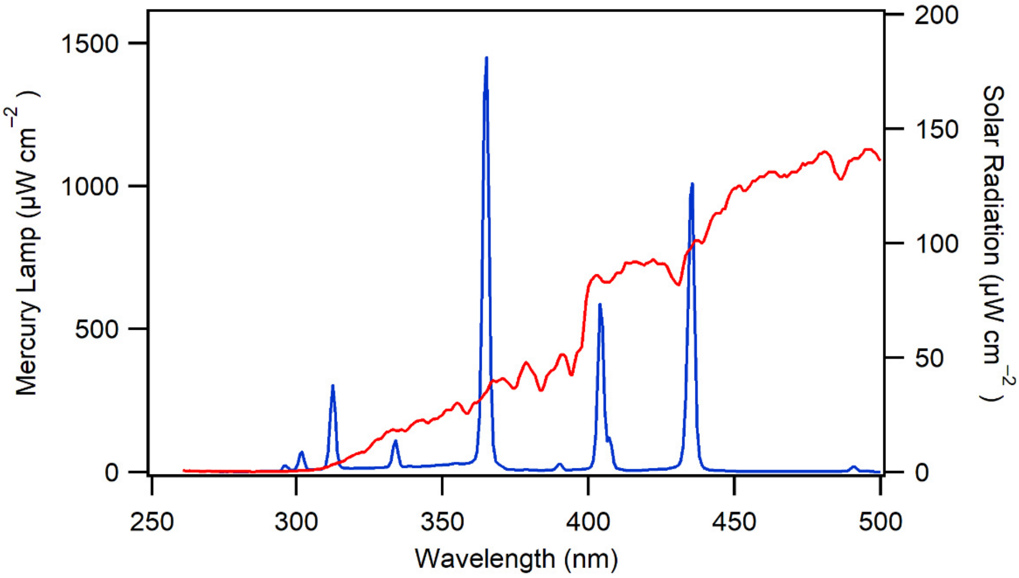

Figure S3. Emission spectrum of a Mercury lamp used in the SEPAP machine (blue line) and Solar radiation arriving at earth (red line).

Size analyses from SEM images

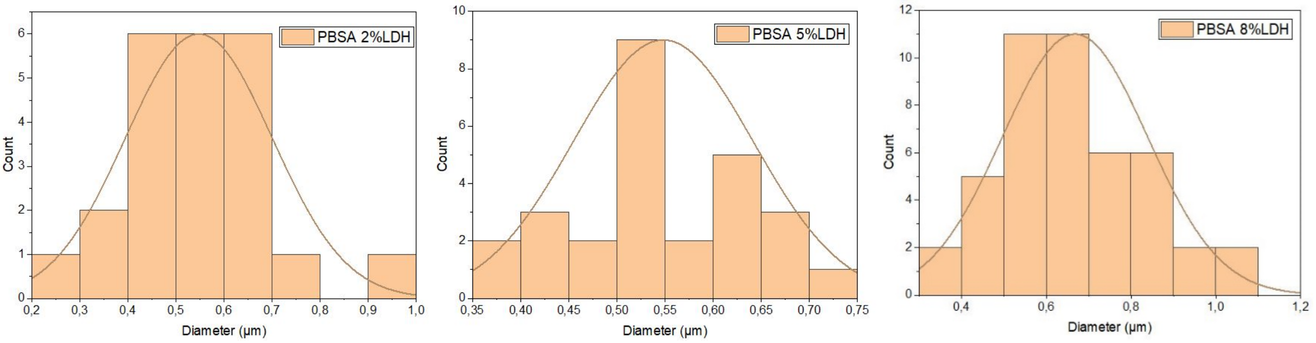

Figure S4. Size distribution (diameter) of detected LDH particulates in SEM images, treated with ImageJ software. The majority (>90%) of the detected particulates are equal or smaller than average SORBACID® 911 particle size (d50) of ≤ 1 μm.

Thermal analyses

Table S1. TGA results of PBSA and PBSA composites.

Sample	Onset of decomposition, °C	Temperature at 50% weight loss, °C	Peak of 1st derivative, °C	Residue at 600 °C, %
PBSA	372.4	398.5	401.8	1.34
PBSA + 2% LDH	338.5	367.8	376.8	5.3
PBSA + 5% LDH	317.3	351.8	360.2	6.1
PBSA + 8% LDH	331.0	356.2	355.2	5.4

Infrared spectroscopy pf PBSA and PBSA-LDH nanocomposite

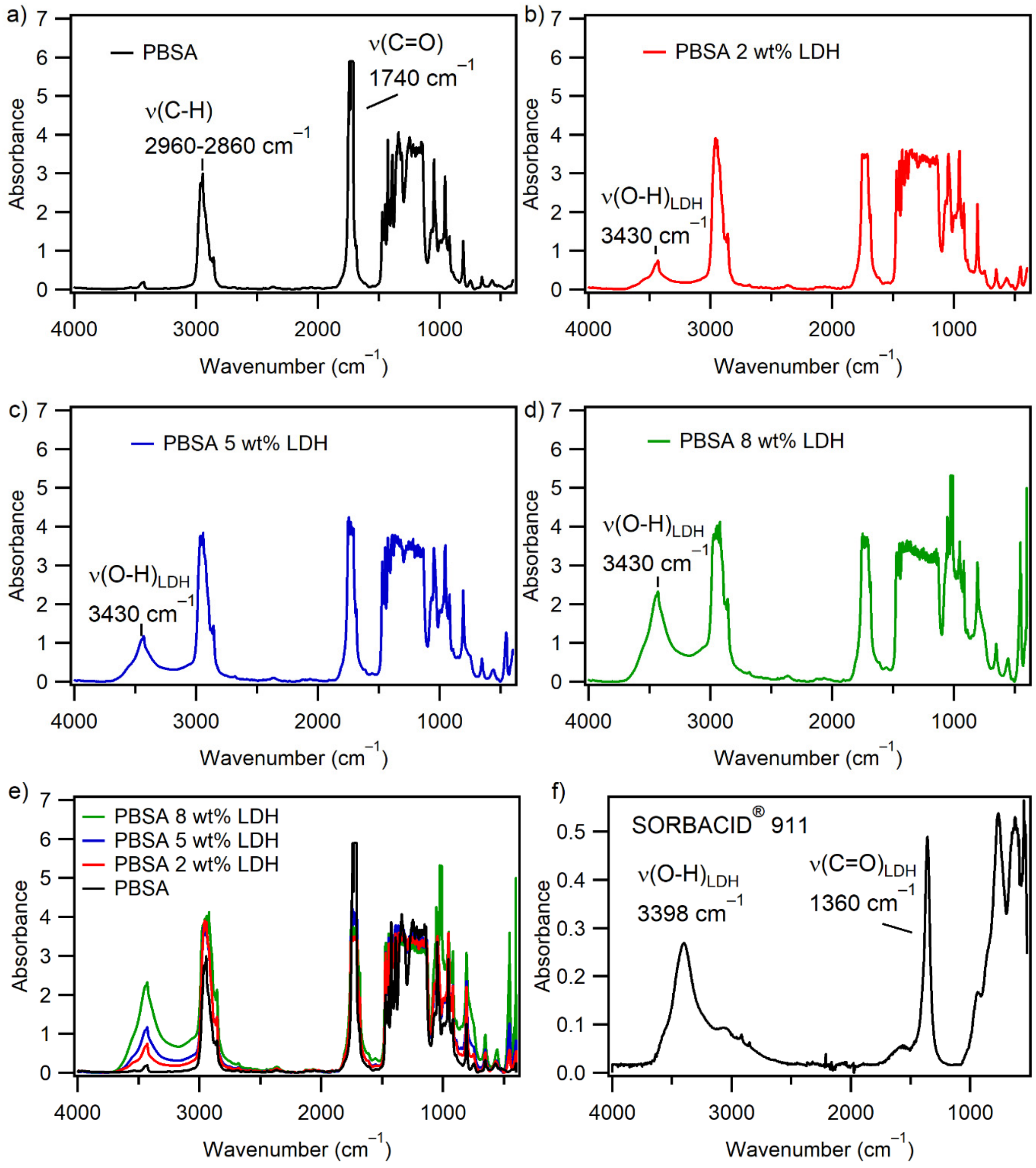

Figure S5. FT-IR spectra of (**a**) PBSA (**b**) PBSA 2 wt% LDH, (**c**) PBSA 5 wt% LDH and (**d**) PBSA 8 wt% LDH. The spectra of neat PBSA and PBSA-LDH nanocomposites are compared in (**e**) and the IR spectrum of SORBACID® 911 is shown in (**f**).

UV-Vis spectra of PBSA-LDH films

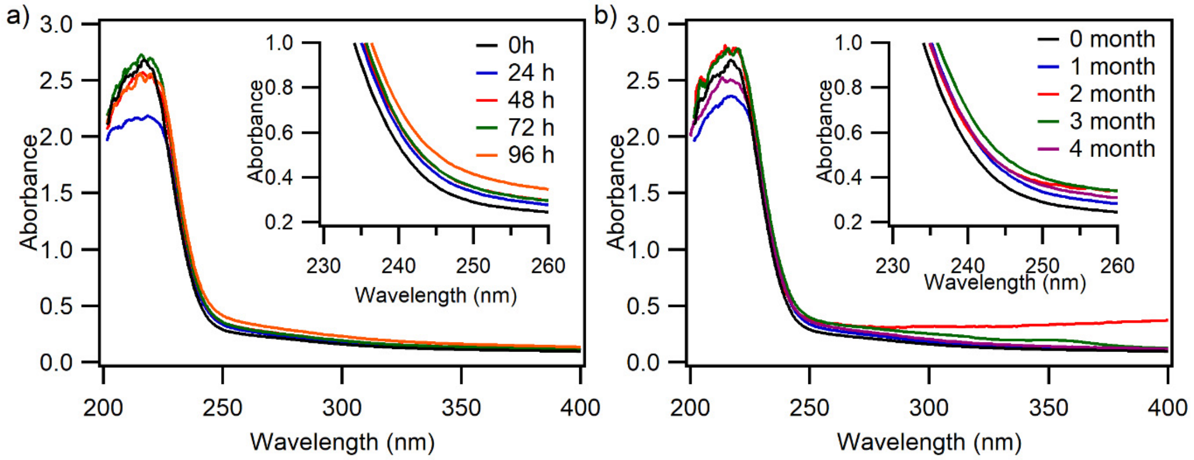

Figure S6. UV-Vis spectra of PBSA before exposure to UV-radiation (black line) and (**a**) after 24 hrs (blue line), 48 h (red line), 72 h (green line), 96 h (orange line) exposure to UV-radiation, and after (**b**) 1 month (blue line), 2 months (red line), 3 months (green line) and 4 months (orange line) of being exposed to natural weathering.

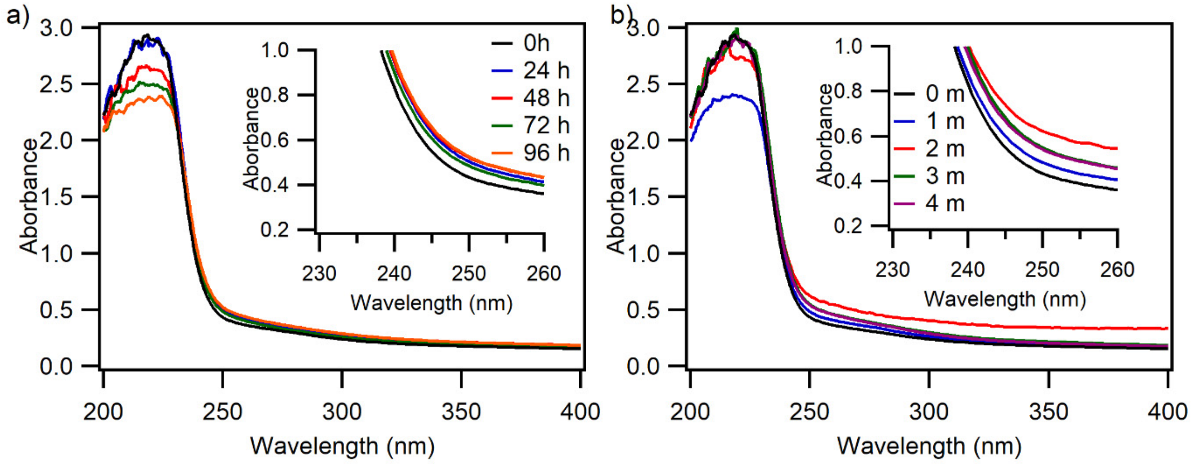

Figure S7. UV-Vis spectra of PBSA + 2% LDH before exposure to UV-radiation (black line) and (**a**) after 24 h (blue line), 48 h (red line), 72 h (green line), 96 h (orange lineexposure to UV-radiation, and after (**b**) 1 month (blue line), 2 months (red line), 3 months (green line) and 4 months (orange line) of being exposed to natural weathering.

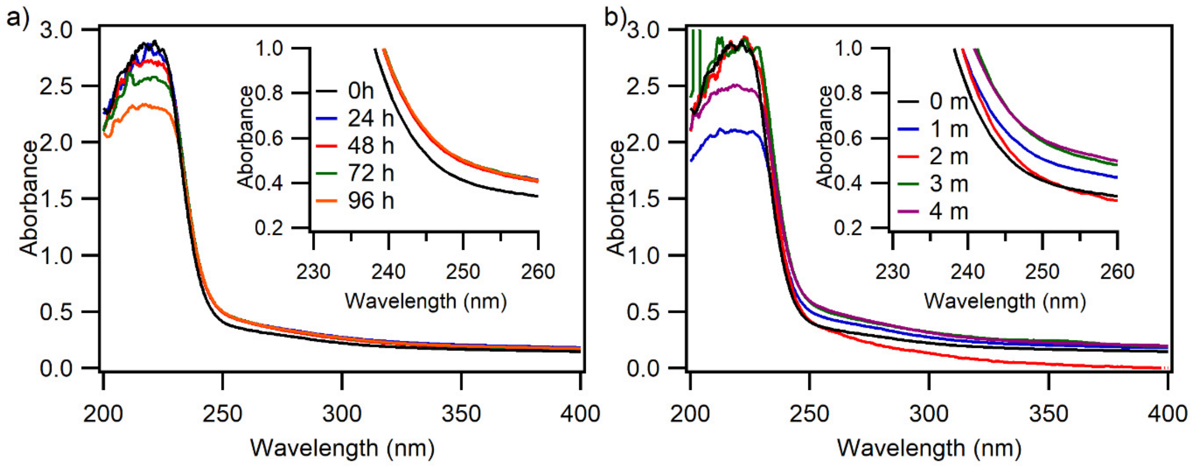

Figure S8. UV-Vis spectra of PBSA + 5% LDH before exposure to UV-radiation (black line) and (**a**) after 24 h (blue line), 48 h (red line), 72 h (green line), 96 h (orange line) exposure to UV-radiation in the SEPAP, and after (**b**) 1 month (blue line), 2 months (red line), 3 months (green line) and 4 months (orange line) of being exposed to natural weathering.

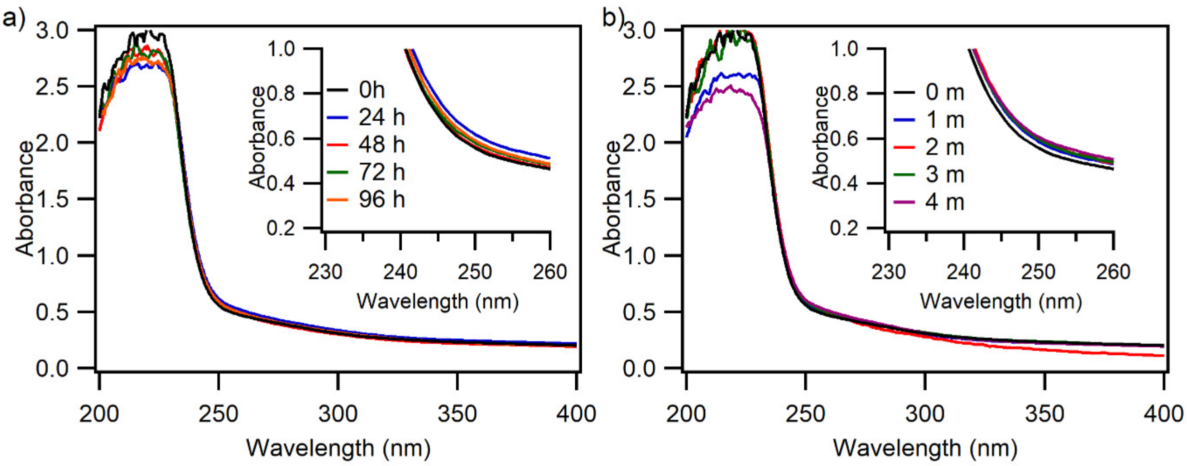

Figure S9. UV-Vis spectra of PBSA + 8% LDH before exposure to UV-radiation (black line) and (**a**) after 24 h (blue line), 48 h (red line), 72 h (green line), 96 h (orange line) exposure to UV-radiation in the SEPAP, and after (**b**) 1 month (blue line), 2 months (red line), 3 months (green line) and 4 months (orange line) of being exposed to natural weathering.

Melt Rheology.

Melt rheology provides a convenient tool to study the competition of chain scissions and recombination reactions occurring through aging of polymers. It is well-known that the zero shear viscosity, η_0. depends on the molecular weight (M_W) and obeys to a power law as described in equation 1[1, 2].

$$\eta_0 \propto M_W^{\propto}, \quad (1)$$

The change in molecular weight of a polymeric material as result of degradation provides valuable information of degradation pathways at a molecular level. By measuring the η_0 over the time of aging of a polymer. The changes in η_0 can indicate whether there is a change in molecular weight of the polymer. A change in the molecular weight as a polymer is aged suggests that a transformation at the molecular level has occurred. leading either to a higher or lower molecular weight. A lower η_0 of a polymer than its initial η_0 corresponds to lower molecular weight. the polymer has been subjected to a chain scission mechanism. Vice versa a higher η_0 correlates to higher molecular weight of the polymer and the polymer has been subjected to a transformation described as a chain recombination mechanism. The η_0 is obtained through the extrapolation of the arc of the circle of a cole-cole model which predicts the variation of the viscosity components (η'' versus η') to be an arc of circle in the complex plane. This is explained more in detail elsewhere by Commereuc and co-workers[1, 2].

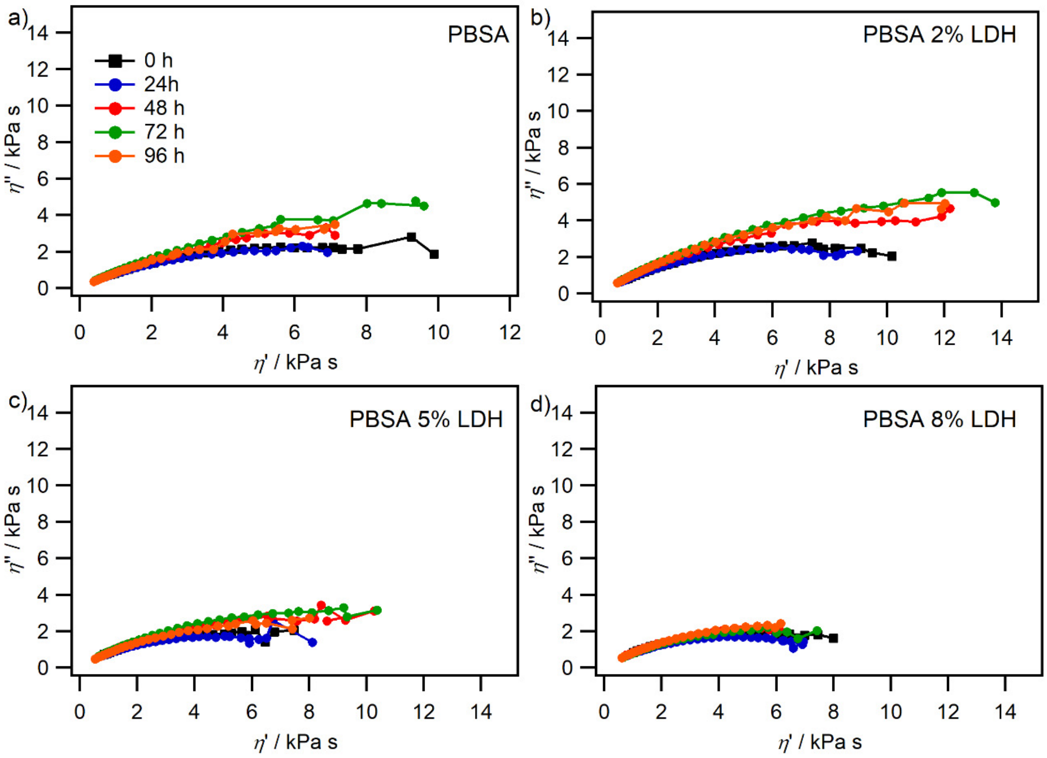

Figure S10. Cole-cole plots of unaged and aged (**a**) PBSA (**b**) PBSA 2 wt% LDH, (**c**) PBSA 5 wt% LDH and (**d**) PBSA 8 wt% LDH in the accelerated photoaging chamber.

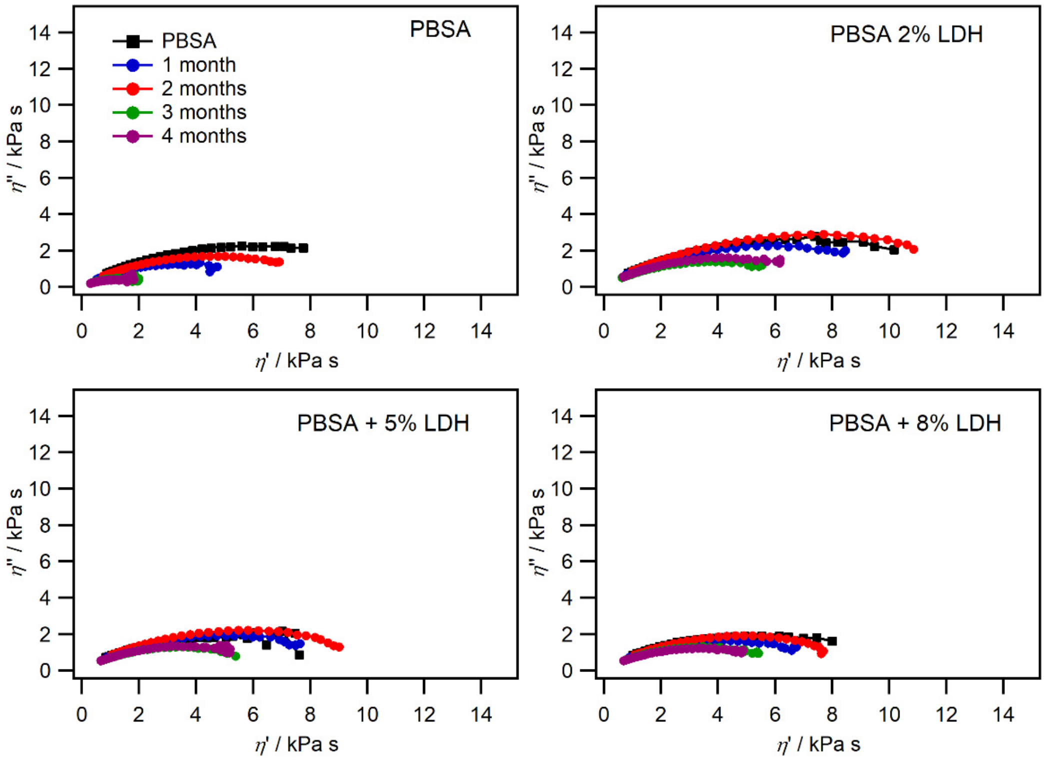

Figure S11. Cole-cole plots of naturally weathered (**a**) PBSA (**b**) PBSA 2 wt% LDH, (**c**) PBSA 5 wt% LDH and (**d**) PBSA 8 wt% LDH.

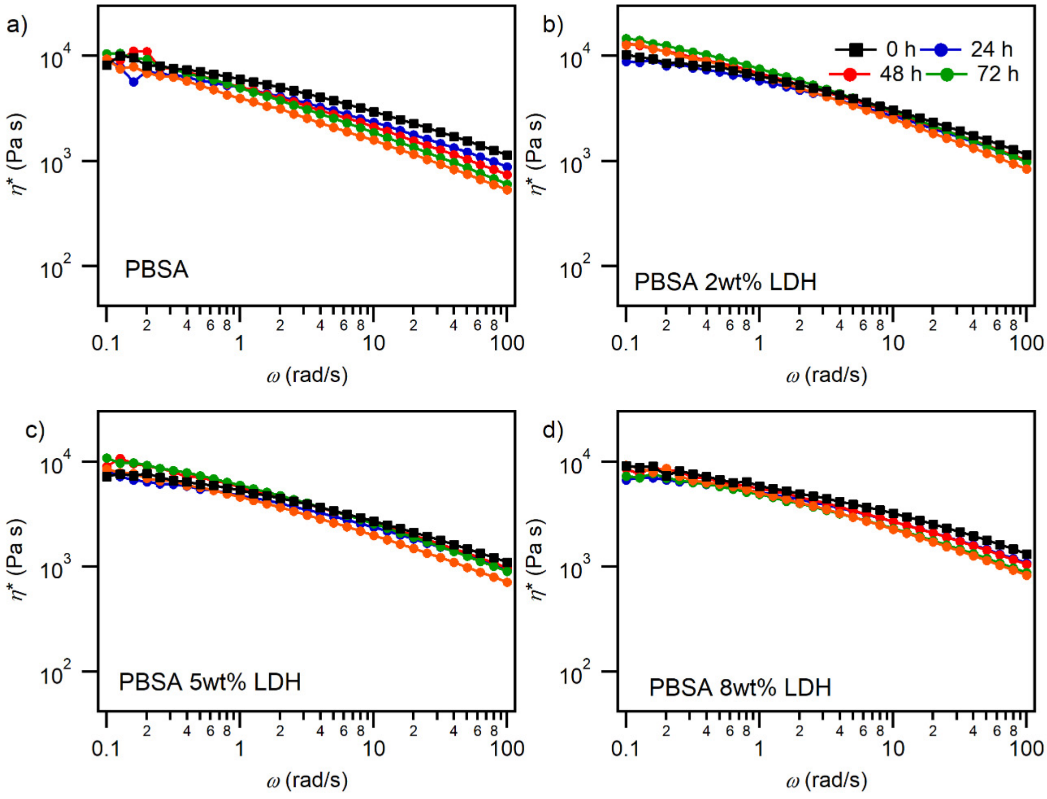

Figure S12. Dynamic viscosity relationship with frequency of unaged and aged (**a**) PBSA (**b**) PBSA 2 wt% LDH, (**c**) PBSA 5 wt% LDH and (**d**) PBSA 8 wt% LDH in SEPAP.

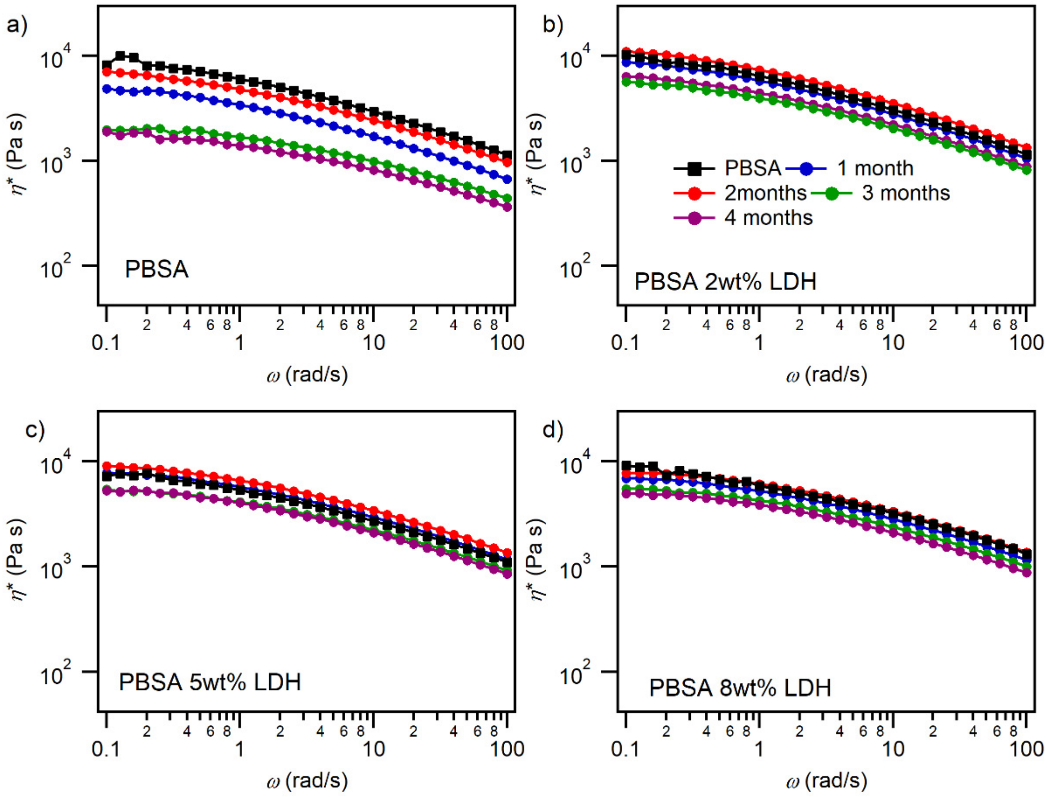

Figure S13. Dynamic viscosity relationship with frequency of naturally weathered (**a**) PBSA (**b**) PBSA 2 wt% LDH, (**c**) PBSA 5 wt% LDH and (**d**) PBSA 8 wt% LDH.
